# Familial adenomatous patients with desmoid tumours show increased expression of miR-34a in serum and high levels in tumours

**DOI:** 10.18632/oncoscience.312

**Published:** 2016-06-30

**Authors:** Sarah-Jane Walton, Amy Lewis, Rosemary Jeffery, Hannah Thompson, Roger Feakins, Eleni Giannoulatou, Christopher Yau, James O. Lindsay, Susan K. Clark, Andrew Silver

**Affiliations:** ^1^ The Polyposis Registry, St Mark's Hospital, Watford Road, Harrow, HA1 3UJ, United Kingdom and Department of Surgery and Cancer, Imperial College London, United Kingdom; ^2^ Centre for Genomics and Child Health and National Centre for Bowel Research and Surgical Innovation, Barts and The London School of Medicine & Dentistry, London, United Kingdom; ^3^ Department of Histopathology, The Royal London Hospital, London, United Kingdom; ^4^ Victor Chang Cardiac Research Institute, Darlinghurst, NSW, Australia; ^5^ The University of New South Wales, NSW, Australia; ^6^ Wellcome Trust Centre for Human Genetics, University of Oxford, Oxford, United Kingdom

**Keywords:** desmoid tumour, familial adenomatous polyposis, microRNA, miRNA-34a-5p, Wnt pathway

## Abstract

Familial adenomatous polyposis (FAP) is rare affecting 1 in 10,000 people and a subset (10%) are at risk of myofibroblastic desmoid tumours (DTs) after colectomy to prevent cancer. DTs are a major cause of morbidity and mortality. The absence of markers to monitor progression and a lack of treatment options are significant limitations to clinical management. We investigated microRNAs (miRNA) levels in DTs and serum using expression array analysis on two independent cohorts of FAP patients (total, n=24). Each comprised equal numbers of patients who had formed DTs (cases) and those who had not (controls). All controls had absence of DTs confirmed by clinical and radiological assessment over at least three years post- colectomy. Technical qPCR validation was performed using an expanded cohort (29 FAP patients; 16 cases and 13 controls). The most significant elevated serum miRNA marker of DTs was miR-34a-5p and *in-situ* hybridisation (ISH) showed most DTs analysed (5/6) expressed miRNA-34a-5p. Exome sequencing of tumour and matched germline DNA did not detect mutations within the miR-34a-5p transcript sites or 3′-UTR of target genes that would alter functional miRNA activity. In conclusion, miR-34a-5p is a potential circulatory marker and therapy target. A large prospective world-wide multi-centre study is now warranted.

## INTRODUCTION

Desmoid tumours (DTs) are rare myofibroblastic, soft tissue tumours. The majority (90%) are caused by mutations in the *CTNNB1* β-catenin gene and occur sporadically [1, 2]. In contrast, the remaining 10% are found in association with familial adenomatous polyposis (FAP), a rare autosomal dominantly inherited condition affecting about 1 in 10,000 people. FAP patients have a germline mutation in one allele of the adenomatous polyposis coli (*APC*) tumour suppressor gene. Mutations of *APC* lead to abnormal activation of the Wnt signalling pathway and tumour formation, notably in the colon and rectum [3, 4].

FAP is characterised by the formation of hundreds to thousands of colorectal adenomas that progress to colorectal cancer in nearly all affected individuals if they do not undergo prophylactic colectomy [5]. Patients with FAP are also at increased risk of other tumours, including duodenal adenomas and carcinomas, and DTs, which have a prevalence of 10–20% in FAP arising with a frequency approximately 850 times that of the general population [6]. DTs are a major cause of morbidity and mortality in FAP patients but can also occur sporadically in the general population [7, 8]. Trauma from prophylactic colectomy (most DTs arise within the first few years after prophylactic colectomy), *APC* germline mutations 3′ of codon 1399 and unknown genetic factors independent of *APC* are significant risk factors for DT development [9, 10].

FAP associated DTs can occur anywhere in the body, although most develop in the small bowel mesentery and abdominal wall, and are locally infiltrative [11]. The majority are found incidentally on cross sectional imaging, or present as a mass, and do not cause significant problems. However, some grow aggressively, invading local surrounding tissues and causing complications including bowel and ureter obstruction, fistulation, abdominal sepsis and even death [7]. There is a low threshold for radiological imaging to assess FAP associated DT development, particularly after surgery, and multiple imaging studies are often required following a diagnosis of intra-abdominal DT to assess growth and accompanying complications [12]. Treatment is difficult, with anti-oestrogens and NSAIDs frequently used as first line therapy, but without any good quality evidence of efficacy. Cytotoxic chemotherapy can be used, but ultimately some patients require surgery, which often results in extensive small bowel resection and long-term parenteral nutrition requirement in complex cases [7].

DTs represent the greatest challenge in the management of FAP and there is a significant unmet clinical need for biomarkers to provide early indication of DTs, particularly the presence of intra-abdominal DTs that may not be obvious at clinical examination, and to predict subsequent aggressive growth. Such markers could also act as an adjunct in determining timing of surgery and reducing frequency of radiological imaging, and in monitoring response to treatment. Investigations to identify biomarkers also have the potential to identify novel targets for therapy. However, large-scale biomarker studies are hampered significantly by the extremely low prevalence of FAP DTs (1/100 000 of the general population). Identifying appropriate controls (FAP patients who do not form DTs) is also clinically challenging, as it is difficult to verify that they represent genuine controls: FAP patients often only form DTs following surgery and are not routinely screened for DTs. Significant resources and follow-up will be necessary to rule-out DT formation in any future prospective trial.

MicroRNAs (miRNAs) are short non-coding RNA molecules that regulate post-transcriptional gene expression and cellular activities including fibrosis and tumour development. Abnormal expression of specific miRNA molecules has been implicated in the development of fibrotic disorders, for example miR-29 in cardiac fibrosis [13] and more recently fibrostenosing Crohn's disease, which is characterised by intestinal fibrosis [14]. MiRNAs also play a role in cancer development and metastasis, functioning as tumour suppressors or oncogenes [15, 16]. Circulating miRNAs can act as non- invasive biomarkers of fibrosis [14, 16] and as novel treatment targets through the use of miRNA antagonists [17]. In this study we test the feasibility and potential utility of circulating miRNAs in serum to act as non- invasive biomarkers of rare FAP-associated DTs, and also assess the levels of the most promising miRNA within the tumours.

## RESULTS

### Patient characteristics

Patients with a diagnosis of FAP with DT and with follow-up at a single institution were identified from the institution polyposis registry database (St Mark's Hospital, Harrow), the largest such registry in Europe, and the second largest in the world. Every diagnosis of DT was confirmed on cross sectional imaging (CT or MRI). Serum samples were obtained from individuals with FAP and DT, and control samples acquired from FAP patients with both clinical and radiological confirmation of DT absence. All control participants were recruited at least 3 years after their prophylactic colectomy, beyond the time when a DT was most likely to be detected [10]. Patient records were analysed to ensure none of the control participants had a previous history of DT. Table 1 compares demographic data between the identified FAP DT cases and FAP controls. The overall median age of participants was 43 years (range 20-72). There was no significant difference between controls and DT formers in terms of gender and age. Unsurprisingly, the proportion of patients with an *APC* mutation 3′ of codon 1399 was significantly higher in the DT group compared to controls (p=0.044). Mutations in this region are a recognised risk factor for DT development [10].

**Table 1:** Demographic data comparing FAP desmoid tumour and FAP controls

	FAP-controls n (%)	DT cases n (%)	p-value
Gender			
Male	7 (54)	11 (69)	0.466*
Female	6 (46)	5(31)	
Age at time of study (median years)	44 (22-72)	38 (20-64)	0.308**
*APC* mutation 3′ 1399			
Yes	1 (8)	7 (44)	0.044*
No	12 (92)	9 (56)	

* Fisher's exact test,

** Mann Whitney-U test

### Comparison of patient serum from DT cases and FAP controls identifies differentially expressed microRNAs

**Figure 1:**
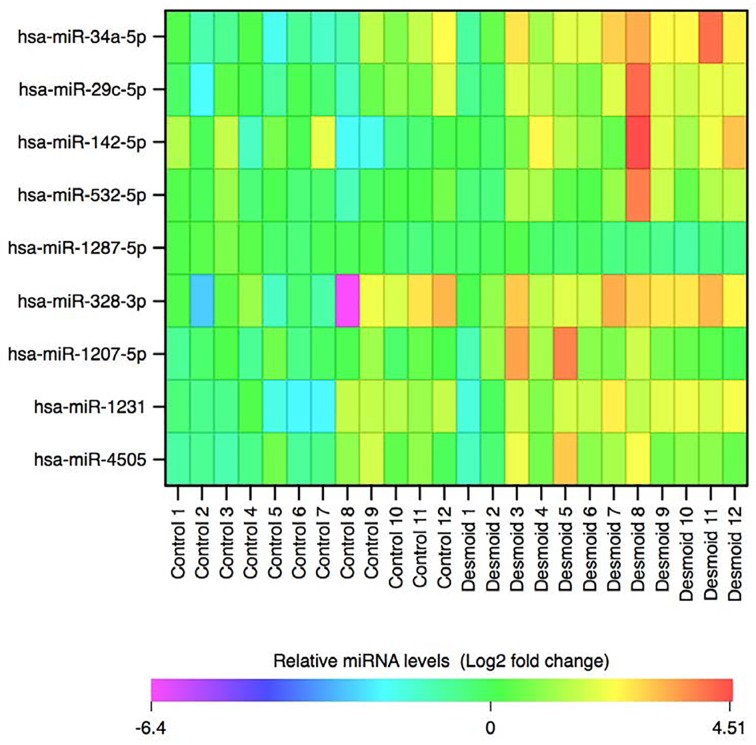
Heatmap comparing serum miRNA levels between desmoid tumour patients and FAP controls. Data are presented as a log2 fold change relative to the mean of FAP controls. The Y axis lists miRNAs altered in the sera of DT patients relative to FAP controls ranked in ascending order according to p-value. The x-axis lists the sample identifiers.

Qiagen determined the levels of 384 miRNAs in the serum of patients with FAP by array profiling. Data were normalised to an exogenous spiked in control probe (Cel- miR-39) and no significant haemolysis was detected in any of the samples (Supplementary Figure 1). Two cohorts of FAP patients (24 patients in total) with and without DTs (n=6 of each in both cohorts) were assessed. Each cohort was processed and analysed independently in two separate batches. Comparison of miRNA levels in serum of FAP controls and DT cases identified a number of differentially expressed miRNAs in each cohort (Table 2 and 3). Only one miRNA, miR-34a-5p, was found to be independently associated with DTs in both cohorts; serum levels of miR-34a-5p were increased in DT patients relative to FAP controls. Moreover, the association of miR-34a-5p with DTs was even stronger when the data for the two cohorts were combined to increase statistical power. In total, the combined analysis identified nine differentially expressed miRNAs, all of which were increased in DTs relative to FAP controls. Of these, miR-34a-4p was the most significant, whilst increased levels of miR-29c-5p were also strongly associated with the presence of DTs in the FAP population (Table 4, Figure 1). However, in this combined analysis statistical significance was lost once a stringent Bonferroni correction was applied to correct for multiple testing.

**Table 2:** miRNAs that differentiate desmoid tumour patients from FAP controls in cohort 1

Mature ID	p-value	Fold change (Log2)
hsa-miR-1287-5p	0.003	−0.464
hsa-miR-34a-5p	0.007	2.288
hsa-miR-532-5p	0.008	1.790
hsa-miR-29c-5p	0.008	1.922
hsa-miR-142-5p	0.010	2.655
hsa-miR-382-5p	0.014	1.324
hsa-miR-143-3p	0.015	2.179
hsa-miR-145-5p	0.022	1.870
hsa-miR-2467-3p	0.030	−0.954
hsa-miR-3120-3p	0.031	1.820
hsa-miR-101-3p	0.033	2.106
hsa-miR-19b-3p	0.035	1.885
hsa-miR-637	0.041	−1.137
hsa-miR-133b	0.042	0.939
hsa-miR-19a-3p	0.044	1.768
hsa-miR-4687-5p	0.045	−1.014
hsa-miR-4688	0.045	−1.198
hsa-miR-4770	0.046	1.465
hsa-miR-503-5p	0.047	1.516
hsa-miR-324-5p	0.047	2.352
hsa-miR-339-3p	0.048	2.139

**Table 3:** miRNAs that differentiate desmoid tumour patients from FAP controls in cohort 2

Mature ID	p-value	Fold change (Log2)
hsa-miR-34a-5p	0.011	1.909
hsa-miR-328-3p	0.014	2.011
hsa-miR-138-1-3p	0.020	1.090
hsa-miR-631	0.021	1.281
hsa-miR-4302	0.033	−0.856
hsa-miR-1231	0.034	1.626
hsa-miR-1207-5p	0.042	1.821
hsa-miR-629-3p	0.046	0.872
hsa-miR-1180-3p	0.048	−0.711

**Table 4:** miRNAs that differentiate desmoid tumour patients from FAP controls in a combined analysis of cohorts 1 and 2

Mature ID	p-value	Fold change (Log2)
hsa-miR-34a-5p	0.001	2.099
hsa-miR-29c-5p	0.003	1.531
hsa-miR-142-5p	0.008	1.584
hsa-miR-532-5p	0.011	1.045
hsa-miR-1287-5p	0.013	−0.369
hsa-miR-328-3p	0.013	2.210
hsa-miR-1207-5p	0.016	1.128
hsa-miR-1231	0.018	1.400
hsa-miR-4505	0.033	0.969

### Altered serum microRNA level profiles in patients with aggressively growing and quiescent desmoids

In a separate sub-analysis of the array data, serum miRNA level profiles within the whole DT population (n=12) were compared between patients with aggressively growing (n=3) and quiescent DTs (n=9). Aggressive tumours were defined as those with evidence of clinical and radiological growth with associated complications, e.g. bowel obstruction, perforation or ureteric obstruction within a six-month period prior to taking the serum sample. Stable tumours were defined as those without clinical or radiological change in tumour growth over the previous 24 months. This comparison identified three miRNAs with an unadjusted p-value ≤0.05. The most significantly differentially expressed miRNA between these two groups was miR-3646, which was decreased in the serum of patients with aggressive DTs (Supplementary Table 1).

### Differentially expressed microRNAs in sera are validated by qPCR

**Figure 2:**
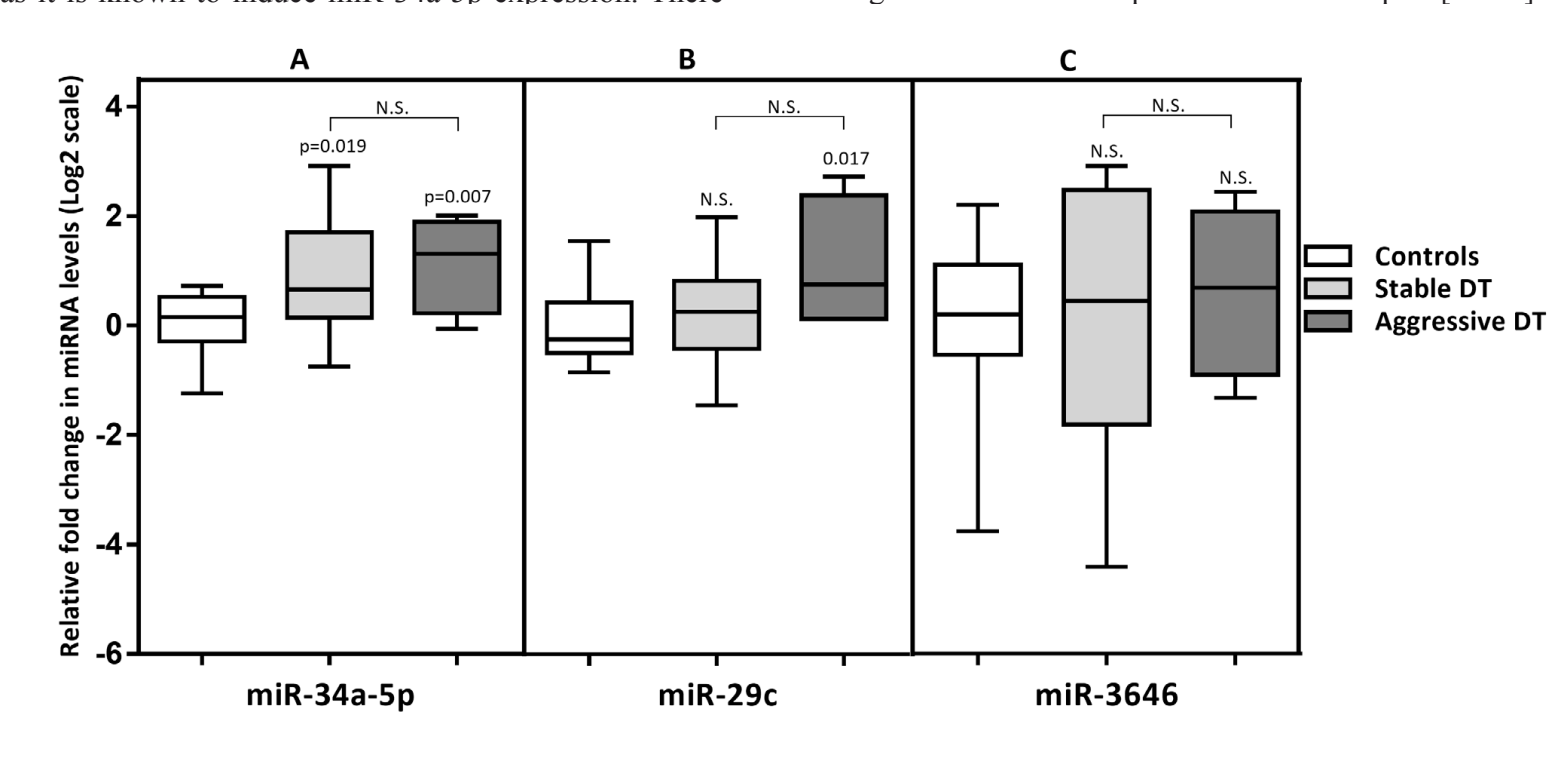
Box and whisker plot comparing serum miRNAlevels in desmoid tumour patients and FAP controls. The p values for comparisons to FAP controls are stated on the graphs. No significant differences between quiescent and aggressive DTs were observed.

To validate the array, miR-34a-5p and miR-29c- 5p were quantified by single qPCR assays as markers of DTs in the FAP population; levels of miR-3646 were also assessed as a marker of aggressive DTs. The qPCR cohort (13 FAP controls and 16 DT cases comprising 12 quiescent DTs and 4 aggressive DTs), although expanded in number, included the samples previously analysed by array. Hence, the qPCR analysis acts as an important technical validation with samples from both array cohorts run in a single batch. As for the array, miRNA levels for this analysis were normalised to an exogenous spiked-in control. Initial analyses identified a statistical outlier in the FAP control group following comparison of miR-34a-5p levels in DTs and FAP control groups (Supplementary Figure 2); this sample was therefore excluded. After adjusting for this single outlier in the control group, greater discrimination was possible between DTs and FAP controls and a significant increase in miR-34a-5p in both quiescent and aggressive DTs was detected in line with the results of the array (p=0.019 and p=0.007, respectively, Figure 2A). In contrast, levels of miR-29c-5p were only significantly elevated in the aggressive DT group compared to FAP controls (p=0.017, Figure 2B). However, contrary to the array findings, the single qPCR assay failed to show any significant difference in the level of circulating miR-3646 between patients with an aggressive DT compared to those with a stable one (Figure 2C).

### MicroRNA *in situ* hybridisation detects substantial miR-34a-5p expression in desmoid tumours

**Figure 3:**
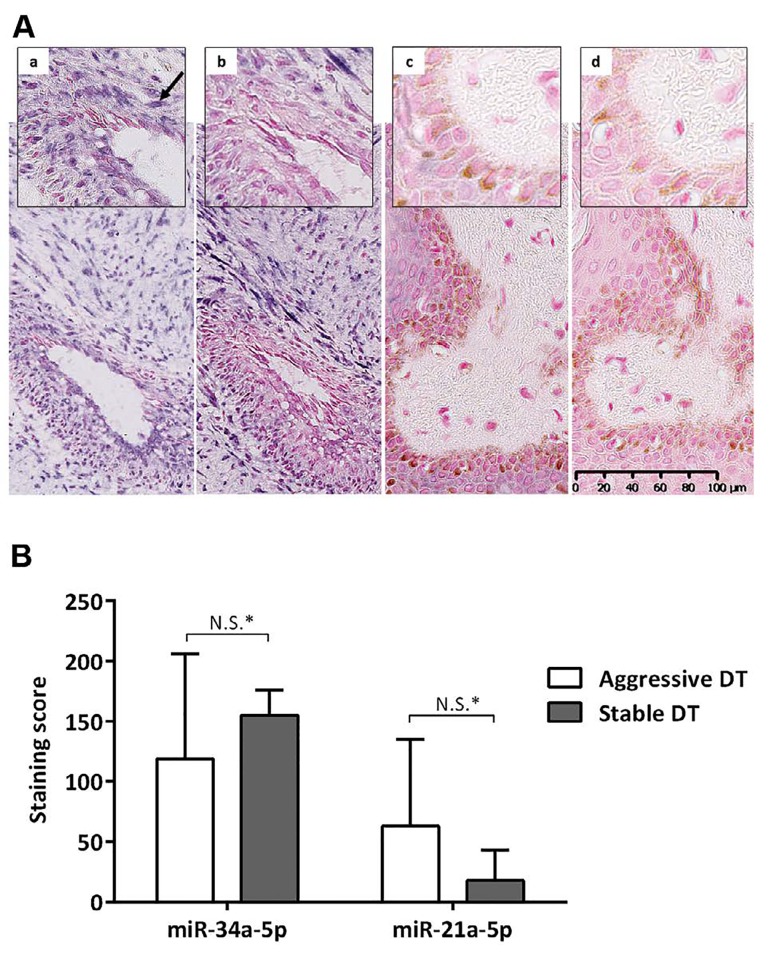
In Situ Hybridisation for miR-34a-5p A. (a) and miR-21a-5p (b) in desmoid tumours and miR-34a-5p (c) and miR-21a-5p (d) in normal skin. High expression of miRNA- 34a-5p and miR-21a-5p seen in the desmoid tumour from patient 1 (Table 5) compared to weak staining both of the skin controls. Purple cytoplasmic staining (black arrow) represents positive staining for the relevant miRNA and pink negative. Magnification x20 and x40. B. Comparison of miR-34a-5p, miR-21-5p mean staining scores in aggressive and stable desmoid tumours. *Mann Whitney-U test

**Figure 4:**
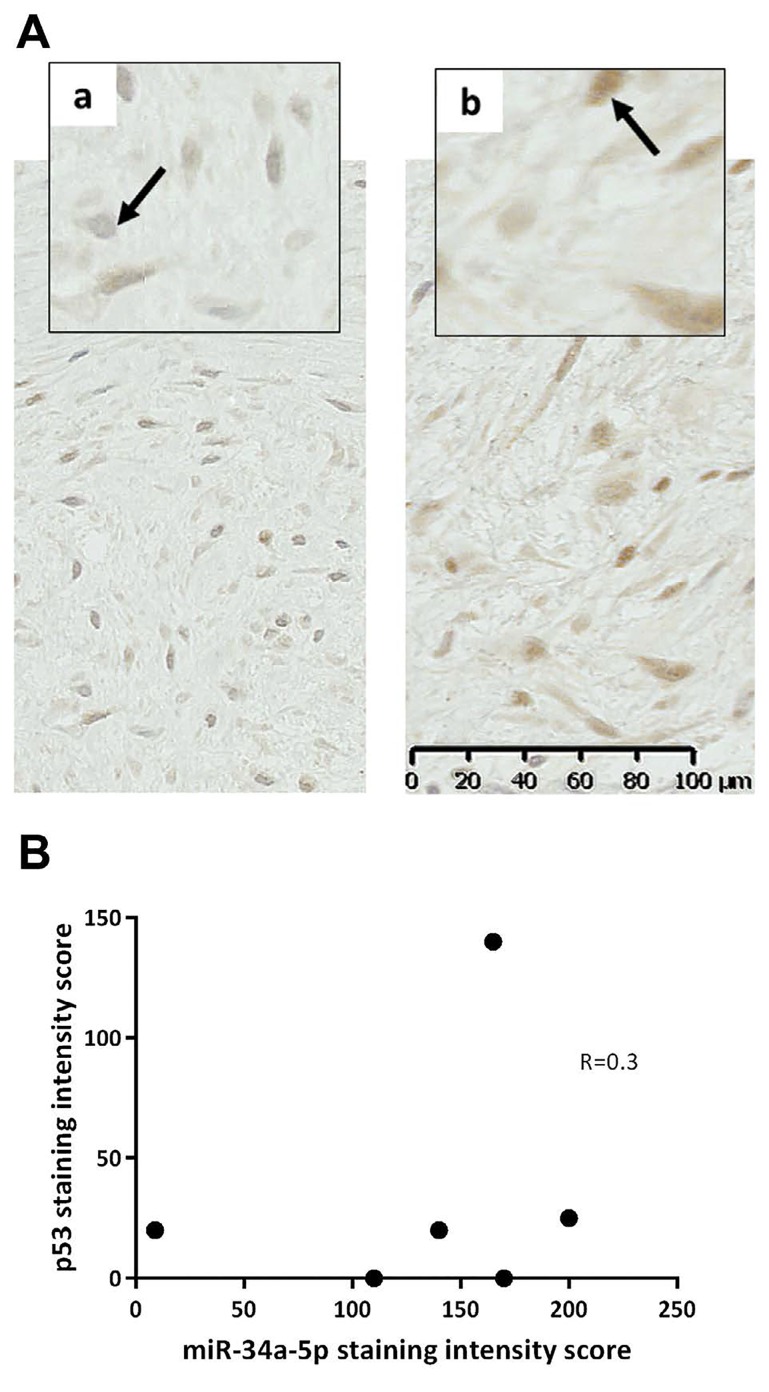
Immunohistochemistry p53 expression in desmoid tumour patients. A. (a). Low weighted score (case 1, supplementary table S2) and (b). high weighted score (case 2). Arrows indicating negative p53 blue staining cells and positive nuclei staining brown. x20 and x40 magnification. B. Correlation between miR-34a-5p and p53 expression in desmoid tumours.

Analysis of serum identified miR-34a-5p as the most significant marker of DT formation. We therefore sought to determine whether miR-34a-5p was expressed within DTs (n=6) using sections cut from FFPE tissue sections and ISH miRNA technology. The expression of miR-34a-5p in DTs was compared to miR-21a-5p, which acted as a positive control for staining, given its reported expression in fibroblasts and involvement in fibrosis [18, 19]. In 5 of the 6 tumours miR-34a-5p was expressed (high expression in 4/5; low expression in 1/5, but close to the arbitrary high/low cut-off; Table 5). Positive staining for miR-21a-5p was also observed in 3/6 DTs (Figure 3A; Table 5), whereas, healthy skin control tissue sections were negative for these probes (Figure 3A). In this small cohort, the intensity of staining for miR-34a-5p and miR- 21a-5p were not correlated with the aggressiveness of the DTs (p=0.8, p>0.99, and p=0.8, respectively) (Figure 3B). Levels of P53 were also investigated in serial sections, as it is known to induce miR-34a-5p expression. There was no nuclear p53 expression in 2/6 tumours analysed, low expression in 3/6 tumours and high expression in one aggressive DT (Figure 4A; Supplementary Table 2). In these sections, there was no direct correlation between p53 staining intensity and miR-34a-5p staining (Spearman's rank correlation coefficient of 0.3, p=0.567) (Figure 4B).

**Table 5:** ISH miR staining scores for aggressive and stable DTs

Phenotype/case	Weighted staining score			
	miR-21-5p		miR-34a-5p	
**Aggressive**	**A**	Level	**A**	Level
**1**	130	high	200	high
**2**	120	high	165	high
**3**	0	none	110	low
**4**	0	none	0	none
**Stable**				
**5**	35	low	170	high
**6**	0	none	140	high

The weighted score is: (1 × the percentage staining at intensity 1) + (2 × the percentage staining at intensity 2) + (3 × the percentage staining at intensity 3). A weighted score of 120 or more is then arbitrarily defined as high level staining.

### No somatic mutations in miR-34a-5p transcript sites or target 3′ UTR in desmoid tumours

Whole exome sequencing was performed in three tumour-germline pairs of DT formers at an average coverage of ∼30x. We interrogated the sequencing data for somatic or loss of heterozygosity mutations within the miRNA transcript sites as well as the binding sites of the miRNA target genes. There were no detected mutations or alterations within the miR-34a-5p transcript sites that would contribute to the altered miRNA activity in DT. We investigated whether somatic mutations occurring in 3′- UTR of the miRNA target genes alter an existing miRNA binding site, interfering with miRNA dependent gene regulation. A list of the miRNA target genes interrogated is provided in the Supplementary Table 3 as predicted by the TargetScan algorithm [20]. We found no mutations in the 3′UTR of target genes of miR-34a-5p that would disrupt the miRNA binding sites. There is therefore no evidence of tumour alterations that would indicate that the tumour is escaping the action of the miRNA. This observation is in agreement with previous studies that suggest that somatically acquired mutations in miRNA genes or their target 3′ UTR are infrequent in tumour samples [21-23].

## DISCUSSION

DTs are the largest challenge in the management of FAP and biomarker studies are invariably hampered significantly by the extremely low prevalence of FAP DTs. We have secured serum and DTs for this study from the St Mark's Polyposis Registry, the largest such registry in Europe, and the second largest in the world. Our study has explored differentially expressed miRNAs in the sera of FAP patients with and without DTs. In this study two independent cohorts of extensively phenotyped patients were analysed, and a technical qPCR validation analysis with all samples run in a single block was performed. This analysis identified increased levels of miR-34a-5p as a marker of DT in the FAP population in both cohorts analysed, and this was confirmed by qPCR. However, the current clinical utility of miR-34a-5p, as a non-invasive biomarker of DTs in the FAP population, is likely limited due to the observed overlap in levels of miR-34a-5p between DT-formers and FAP controls.

Altered tissue miRNA profiles within largely sporadic DTs have previously been associated with aggressive DTs [24]. In this study the potential of serum miRNA markers for identification of aggressive DTs was therefore also explored. Analysis of the array data identified only three miRNAs that were altered in aggressive DTs relative to quiescent tumours; of which, miR-3646 was the most significant. However, due to the relatively low number of aggressive DTs analysed (n=3) and the high number of miRNAs analysed all statistical significance was lost once the p-values were adjusted for multiple testing. Moreover, difference in miR-3636 could not be validated by qPCR. The low validation frequency for miRNA markers of tumour aggressiveness likely reflects the comparatively low number of aggressive DTs analysed. The low number of samples highlights the difficulty in obtaining well phenotyped samples (both controls and cases) for rare diseases such as FAP; FAP patients with DTs are an even rarer group of patients (1/100 000 of the general population). Unfortunately, additional samples would require a major collaborative effort across FAP management centres world-wide to combine expertise and resources to maximise sample numbers. Nevertheless, given the severity and refractory nature of the disease biomarker investigations are of importance: potential applications of non-invasive biomarkers of DT could include earlier detection, and identification of more aggressive tumour phenotypes to enable swift institution of medical therapy. Early identification of DT patients would also prompt closer follow-up, particularly for aggressive DTs. A reliable biomarker that reflected DT growth could also reduce the frequency of radiological imaging to assess stable tumours. The findings of this study should provide the impetus for further research in this area and in fibrosis in general.

Attempts to study miRNAs levels in DT tissue were also made in this study. There are a number of difficulties in quantitatively assessing expression levels in DTs, not least the lack of an appropriate control tissue and DT cell lines. In this study miRNA levels in DT tissue were investigated using miRNA ISH techniques. This analysis demonstrated that miR-34a-5p is highly expressed by DTs relative to other miRNAs known to be involved in cancer and fibrosis e.g. miR-21a-5p. In this small cohort, the intensity of miR-34a-5p staining in DTs was not correlated with the aggressiveness of the tumour, nor were they correlated with P53 levels, which are known to directly induce miR-34a-5p expression in other systems [25]. Moreover, exome sequencing did not detect any mutation in the transcription sequence of miR-34a-5p or in the 3′UTRs of target mRNAs that might disrupt this miRNAs activity in DTs.

These data raise two important possibilities; one, that miR-34a-5p has a functional role in the growth of these tumours, and two, that the increase in miR-34a-5p in the serum may originate from the DT.

**Figure 5:**
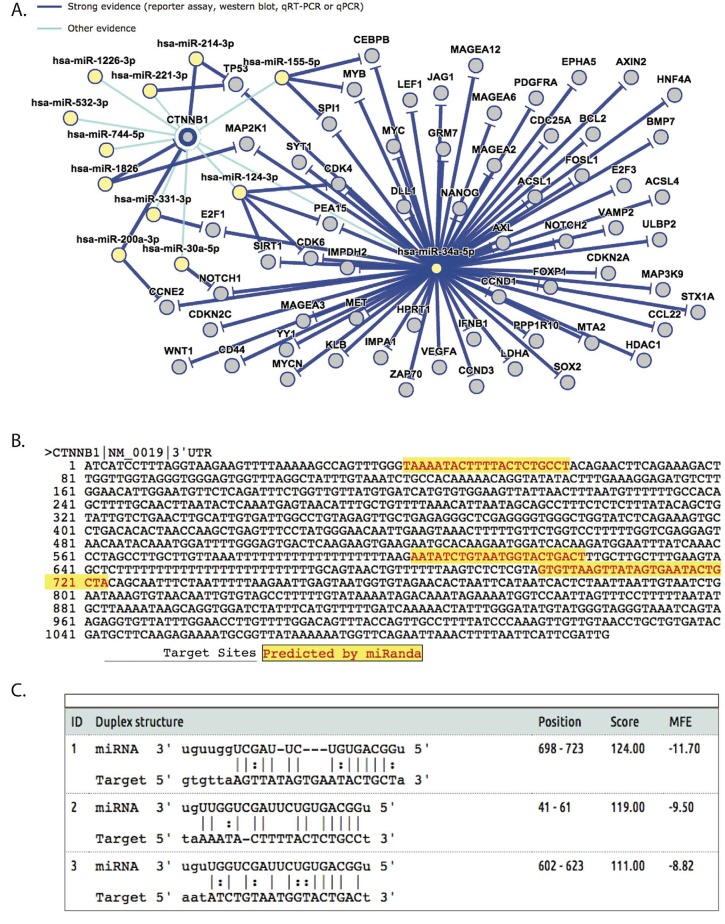
Interaction between miR-34a-5p and CTNNB1 as determined by miRtarBase. A. Network constructed by miRtarBase showing the interaction between genes and miRNAs, B. Potential binding sites of miR-34a-5p in CTNNB1 3′UTR as predicted by miRanda and displayed by miRtarBase. C. miRNA-target interactions as predicted by miRanda and provided by miRtarBase. The alignment scores as well as a Minimum free energy (MFE) values that indicate the strength of the prediction are also shown.

Targets of miR-34a include several genes in the Wnt signalling pathway, which is integral for DT development. For example, the p53/miR-34 axis is known to suppress the canonical Wnt signalling pathway. Conversely, the Wnt signalling pathway can induce miR-34 expression [26] suggesting the existence of a feedback loop. Direct targets of miR-34a-5p also include *CNNTB-1*, with the miRanda prediction algorithm, indicating 3 potential binding sites in the 3′UTR of *CTNNB1* (Ref Seq transcript *NM_0019*) where miR-34a-5p could be binding (Figure 5). Among other experimentally validated target genes, LEF1 a key transcription factor in the Wnt signalling pathway is negatively regulated by miR-34a [27, 28].

With respect to circulating miR-34a-5p, a few studies have reported on serum levels and association with tumours including breast cancer, gastric cancer and lymphoma. In contrast to the reduced expression seen in many tumours, these studies found an upregulation of miR-34a in the serum of affected patients. Roth et al found an increase in circulating miR-34a in those patients with metastatic breast cancer compared to healthy controls [29]. Similarly, an increase was seen in patients with gastric cancer [30] although a reduced serum miR-34a level was reported in lymphoma [31]. Reduced expression in malignant tumour tissue contrast with the high expression of miR-34a-5p observed in DT tissue in this study. However, as DTs are not invasive tumours but benign myofibroblast tumours, it is possible that miR-34 has a role in driving fibrogenesis in DT patients. Compared to our knowledge on the role of miR-34 in malignant tumours, much less is known about the function of this miRNA in the context of fibrosis. However, In support of this hypothesis, up-regulation of miR-34 has been also associated with a number of fibrotic conditions including liver cirrhosis [32], myocardial infarction and fibrosis [33, 34] and neuro-degenerative disorders [35]. Also, increased circulatory levels of miR-34a-5p were associated with intestinal fibrosis in Crohn's disease using the same array platform [16].

Our study has provided proof-of-principle that miRNA biomarkers for DT management are a possibility. miR-34a-5p is a novel and lead candidate following validation of increased expression in two independent cohorts of serum and its elevated expression in tumours. Since a validated candidate has not been indicated before, a better understanding of the role of miR-34a-5p and its role in fibrogenesis is urgently required given the recent development of MRX34, a miR-34 analogue now in phase 1 clinical trial for the treatment of cancer [36, 37] and the existence of a miR-34 inhibitor [38]. The potential for off-target side effects of miRNA analogues also need careful consideration given that each miRNA is predicted to target multiple gene targets. Animal models and/or cell culture systems for DTs will be required to determine the functional significance of miR-34a-5p in DT development, although currently none exist.

In conclusion, our study found upregulation of miR-34a-5p in the serum of DT patients relative to FAP controls, supporting its role as a non-invasive biomarker for DT. High levels of miR-34a-5p were also observed in the tumour tissue. These pathways have not been explored before in DT development. This miRNA may offer new treatment target pathways for future research into these rare and complex tumours.

## MATERIALS AND METHODS

### Ethics statement

The study had appropriate ethical approval from London NRES committee and all participants gave their informed consent for a single blood test. Archival formalin-fixed paraffin-embedded (FFPE) DT tissue removed at surgery was obtained for assessment of miRNA expression using *in-situ* hybridisation (ISH) and to assess possible pathway targets, using immunohistochemistry (IHC).

### Serum collection

6ml of whole blood was collected in an EDTA bottle for each participant. Samples were centrifuged at 3000rpm for 10 mins within 1 hour of collection to achieve serum separation. Serum was then removed and stored in a cryovial at −80°C until required.

### MicroRNA Arrays

RNA was extracted from 200 μl of serum and profiling of miRNAs performed by Qiagen (Germany) as previously described [14]. Quantification of serum miRNAs was achieved through SYBR-qPCR using miScript miRNA PCR 384HC array for serum (MIHS- 3106Z). The miRNA array panel set was selected from a previous panel used to investigate fibrosing Crohn's disease [14]. Each array contained a panel of 384 primer sets for thoroughly researched miRNAs known to be highly expressed in serum and/or associated with altered expression in the context of other diseases. To enhance the detection of miRNAs present at low levels in serum, the arrays were performed using a pre-amplified cDNA set with a maximum cycle threshold (Ct) of 30 for all samples and miRNAs. An exogenous spiked in control (Cel-miR- 39-3p) was used for normalisation using the 2^−ΔCt^ method and the data log2 transformed prior to statistical analysis to stabilise for variance and achieve a normal distribution. The delta Ct for miR-23 and miR-451a was used as a haemolysis indicator for the array samples; a value >7 indicated significant risk of haemolysis [39].

### Validation using single qPCR sera assays

Qiagen miRNeasy Serum/Plasma kit (Cat. No. 217184) was used to extract RNA from 200 μl of serum and a synthetic RNA (Ce_miR-39_1) spike was added to enable normalisation of data. RNA was reversed transcribed and qPCR performed as described [16]. Each reaction was performed in duplicate and melt curve analysis undertaken to confirm a single PCR product. Levels of sera miRNA were normalised to corresponding values of Ce_miR-39_1 (MS00019789) spike using the 2^−ΔCt^ method. These normalised data were then log2 transformed prior to statistical analysis.

### MicroRNA In-situ hybridisation

The level of miRNA in DT FFPE tissue was investigated using miRNA ISH technology (Exiqon, Denmark). ISH probes were selected based on the sera PCR results and 4 μm sections from FFPE tissues were sectioned under RNAse clear conditions. A FFPE skin control (from a healthy patient without FAP) was also obtained and sectioned. To ensure adhesion, sections were heated on slides at 65°C for 10 mins before deparaffinising in xylene for 10 mins and hydrating in RNAse-free H_2_O. Sections were digested with 10ug/ ml proteinase K in PBS at 37°C for 30 minutes, washed in RNAse-free H_2_O and dehydrated through increasing concentrations of ethanol (70%, 95% and absolute), 1 min each and then air-dried. Sections were then incubated with Double-DIG (digoxigenin) labelled Locked Nucleic Acid (LNA) ISH probes complementary to the selected miRNAs at 56°C for 2 hours. ISH probes were diluted from a 10 μM stock concentration. Dilutions used for miR-34a-5p and miR-21-5p LNA ISH probes were 1:450 and 1:240, respectively. A scrambled miRNA probe was used as a negative control at a dilution of 1:300. Slides subsequently underwent stringency washes at 56°C: once 5X Saline sodium citrate (SSC) buffer, twice in 1X SSC, twice in 0.2X SSC and finally once in 0.2X SSC at room temperature. Slides were placed in blocking solution (1% triton, bovine serum albumin (BSA) in TBS) for 10 min. Anti-digoxigenein antibody (150U, Roche, Switzerland) at a 1:300 dilution (1% triton, BSA in TBS) was applied to the sections for 45 mins at room temperature, followed by TBS wash and rinse in RNAse-free H_2_O. Nitroblue tetrazolium and 5-bromo-4-chloro-3-indolyl phosphate (NBT/BCIP) reagent (Roche) was applied to detect signal overnight at 4°C. Slides were washed briefly in tap water prior to counterstaining with nuclear fast red, dehydrating in increasing concentrations of ethanol and mounting in Eukitt (Fluka, Germany).

### P53 immunohistochemistry

FFPE 4 μm tumour sections were dewaxed in xylene and placed in absolute alcohol before application of an endogenous peroxide block for 10 mins and rehydrating through graded alcohol concentrations. Antigen retrieval was achieved by microwaving sections in a citrate buffer (pH 8) for 20 mins. Non-reactive staining was blocked using rabbit serum (1:25 dilution) before p53 primary mouse antibody application (1:50, DAKO) for 45 mins. Sections were washed before the secondary rabbit anti- mouse antibody (1:250) was applied for 45 mins. After further washing, antibody binding was detected using a diaminobenzidine reaction kit (kit K3468, DAKO, USA).

### Tissue imaging and scoring

IHC and ISH slides were analysed using a light microscope and scored by a pathologist according to stain intensity and proportion of positively staining cells. The percentage of neoplastic cells showing cytoplasmic staining at each of three levels of intensity (1: weak; 2: moderate; 3: strong) was determined for each marker. This yielded a total percentage of cells staining and enabled the calculation of a weighted score, where higher weight was given to higher intensities of staining. The weighted score was: (1 × the percentage staining at intensity 1) + (2 × the percentage staining at intensity 2) + (3 × the percentage staining at intensity 3). On the basis of the weighted scores, cases were then defined as having high level, low level or no staining.

### Analysis of whole exome sequencing datasets of desmoid tumour samples

Tumour and germline DNA from three paired tumour-germline samples was isolated using standard methods from fresh frozen tumour tissue and peripheral blood leukocytes. Target exonic regions were captured using the Illumina Nextera rapid exome library prep kit (Illumina, UK). The captured regions were sequenced using Illumina HiSeq 2000. The sequence reads were aligned to the human genome reference using Burrows- Wheeler Aligner (BWA) [40]. Tumour specific point mutations were detected by comparing tumour DNA to its paired germline using Varscan2 [41] and were subsequently annotated using ANNOVAR [42]. Mean sequencing coverage was calculated using the total number of reads in called positions for all tumour and germline samples. Tumour specific mutations were excluded if they were covered by less than 10 reads in either tumour or germline sample or had a somatic p-value of <0.05. The TargetScan predictive algorithm [20] was used to determine whether somatic mutations detected in 3′UTRs of miRNA target genes occurred within the miRNA biding sites. Supplementary Table 1 contains the list of target genes of the miRNAs (miR-34a-5p) as predicted by TargetScan v6.2.

### Statistical analysis

Statistical analysis of miRNA array results was performed using the log2 transformed data. For comparison of demographic data and miRNA array discovery analysis, a two-tailed t test was used to determine differences between the two means, with alpha set to 0.05. For subsequent qPCR validation of differences between groups a one-tailed t test was applied.

## SUPPLEMENTARY MATERIALS FIGURES AND TABLES





## ABBREVIATIONS

DTs: Desmoid tumours
FAP: familial adenomatous polyposis
miRNA: MicroRNAs
*CTNNB1*: β-catenin gene
*APC*: adenomatous polyposis coli
FFPE: formalin- fixed paraffin-embedded
ISH: in-situ hybridisation
IHC: immunohistochemistry
LNA: Locked Nucleic Acid
SSC: saline sodium citrate
BSA: bovine serum albumin
TBS: Tris-buffered saline.
