# Supplementary material for: Familial adenomatous patients with desmoid tumours show increased expression of miR-34a in serum and high levels in tumours
Source: Oncoscience. 2016 Jun 30;3(5-6):173–85. doi: 10.18632/oncoscience.312 (PMC4965260; doi:10.18632/oncoscience.312)
Supplement: Supplementary file 1 [file oncoscience-03-173-s001.pdf]

SUPPLEMENTARY MATERIALS

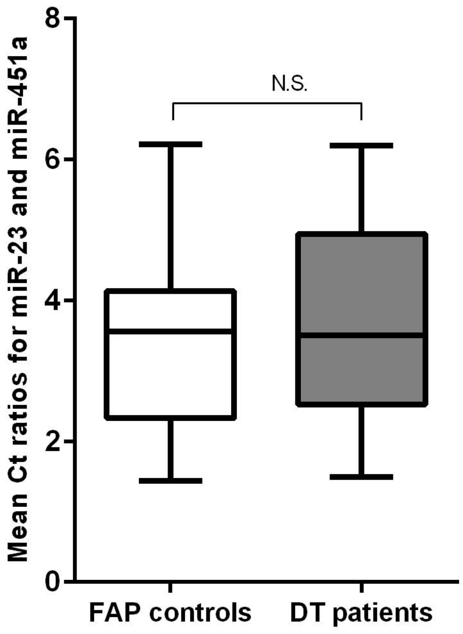

Supplementary Figure S1: Box and whisker plot comparing FAP controls and DT patients mean delta Ct ratios for miR-23 and miR-451a (indicator of haemolysis) for the samples analysed by array. No significant haemolysis was detected in any of the samples; moreover the delta Ct values were similar in FAP patients with and without DTs

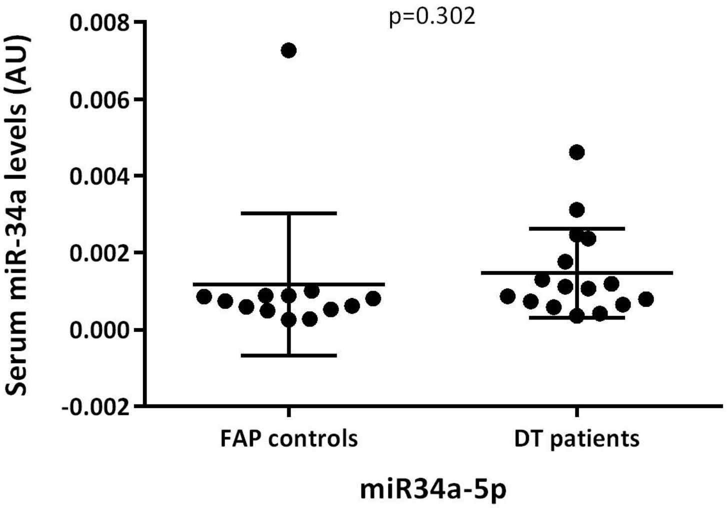

Supplementary Figure S2: Comparison of miR-34a serum levels between FAP controls and DT patients.

Supplementary Table 1: miRNAs that differentiate aggressive from stable DTs in patients with FAP.

Mature ID	p-value	Fold change (Log2)
hsa-miR-3646	0.015	-0.998
hsa-miR-3651	0.034	-1.364
hsa-miR-138-1-3p	0.047	-1.127

Supplementary Table 2. p53 immunohistochemistry staining scores for aggressive and stable DTs. The weighted score is: (1 x the percentage staining at intensity 1) + (2 x the percentage staining at intensity 2) + (3 x the percentage staining at intensity 3). A weighted score of 120 or more is then arbitrarily defined as high level staining.

Phenotype/case	Weighted staining score	
**Aggressive**	**A**	**Level**
**1**	25	low
**2**	140	high
**3**	0	none
**4**	20	low
**Stable**		
**5**	0	none
**6**	20	low

Supplementary Table 3: miR-34a-5p target genes as predicted by TargetScan v6.2 Please see separate Supplementary Table file.
